# Incidental Unruptured Intracranial Aneurysms Do Not Impact Outcome in Patients With Acute Cerebral Infarction

**DOI:** 10.3389/fneur.2021.613027

**Published:** 2021-04-26

**Authors:** Xuan Wu, Zuowei Duan, Yihui Liu, Changwu Zhou, Zhiyun Jiao, Yi Zhao, Tieyu Tang

**Affiliations:** ^1^Department of Neurology, Affiliated Hospital of Yangzhou University, Yangzhou, China; ^2^Department of Medical Imaging, Affiliated Hospital of Yangzhou University, Yangzhou, China

**Keywords:** Unruptured intractanial aneurysms, acute cerebral infarction, outcome, age, diabetes, ischemic stroke history, baseline NIHSS score

## Abstract

**Background:** This study was to examine the patients with acute cerebral infarction (ACI) treated at a single center over 9 years and who underwent Unruptured intracranial aneurysm (UIA) screening by three-dimensional time-of-flight magnetic resonance angiography (3D-TOF-MRA), and to explore the factors associated with outcomes.

**Methods:** The outcome was the modified Rankin scale (mRS) score at 90 days after stroke onset. The outcome was classified into a good outcome (mRS score of 0–2 points) and poor outcome (mRS score of 3–6 points).

**Results:** UIAs were found in 260 (6.5%) of 4,033 patients with ACI; 2,543 (63.1%) had a good outcome, and 1,490 (36.9%) had a poor outcome. There was no difference in outcomes between the two groups (*P* = 0.785). The multivariable analysis showed that age (OR = 1.009, 95%CI: 1.003–1.014, *P* = 0.003), diabetes (OR = 1.179, 95%CI: 1.035–1.342, *P* = 0.013), ischemic stroke history (OR = 1.451, 95%CI: 1.256–1.677, *P* < 0.001), and baseline NIHSS score (OR = 1.034, 95%CI: 1.018–1.050, *P* < 0.001) were independently associated with the 90-day outcomes in patients with ACI. The presence of incidental UIA was not associated with outcomes after ACI.

**Conclusions:** Age, diabetes, ischemic stroke history, and baseline NIHSS score were independently associated with the early outcomes of patients with ACI.

## Introduction

Stroke is characterized by an ischemic infarction of a brain area (ischemic stroke) or an abnormal collection of blood within the brain or the cranium (hemorrhagic stroke), resulting in a focal injury to the central nervous system (CNS) and acute neurological dysfunction (1). Ischemic strokes (80–87% of strokes) usually result from cardioembolism (often from atrial fibrillation), large artery atherosclerosis leading to occlusion, occlusion of small vessels (seen in lacunar strokes), or systemic hypoperfusion (1). On the other hand, hemorrhagic strokes may result from a ruptured aneurysm, hypertension, or secondary to anticoagulation, leading to intracerebral hemorrhage (ICH) or subarachnoid hemorrhage (SAH) (1).

The estimated global incidence of stroke is 2–3 per 1,000 person-years, with older patients and patients with carotid artery stenosis or atrial fibrillation having the highest risk (1). In China, cerebrovascular diseases have become the leading cause of death, and the mortality rate is increasing each year. In 2015, the incidence of acute cerebral infarction (ACI) in China reached 47.0 per 100,000 people (2).

Unruptured intracranial aneurysm (UIA) can be found incidentally during imaging examinations of the head. The mortality rate of subarachnoid hemorrhage caused by the rupture of intracranial aneurysms is up to 30–50% (3, 4). Nevertheless, the clinical management of incidentally identified UIA is highly controversial (5–9), especially for patients with ACI (9–11). Since the acute phase of patients with ACI needs special treatment such as emergency thrombectomy, intravenous thrombolysis, and antithrombotic, the presence of UIA will inevitably increase the complexity of the acute-phase disease management. In addition, there are only clinical observations of small numbers of patients about how comorbidities can affect the early outcomes of cerebral infarction (12, 13).

Therefore, the aim of this retrospective study was to examine the patients with ACI treated at a single center over 9 years and who underwent UIA screening by three-dimensional time-of-flight magnetic resonance angiography (3D-TOF-MRA), and to explore the factors associated with outcomes. The results could provide a basis for further clinical multicenter randomized controlled trials.

## Materials and Methods

### Patients

This retrospective study was carried out in patients treated from January 2009 to August 2018 at the Affiliated Hospital of Yangzhou University. This work has been carried out in accordance with the Declaration of Helsinki (2000) of the World Medical Association. This study was approved by the ethics committee of the Affiliated Hospital of Yangzhou University (approval No. 2018-YKL-015). This article is a retrospective study. Therefore, the Institution waived the requirement to obtain distinct written informed consent from the patients.

The inclusion criteria were as follows: (1) 20–90 years of age; (2) modified Rankin scale (mRS) score ≤ 2 points before the onset of ACI; (3) admission within 24 h after onset; (4) diagnosis was consistent with the China Acute Ischemic Stroke Diagnosis and Treatment Guidelines 2014 (14); (5) underwent magnetic resonance imaging (MRI) and 3D-TOF-MRA examination within 72 h after admission; and (6) complete data. The exclusion criteria were as follows: (1) history of subarachnoid hemorrhage, brain trauma, intracranial hemorrhage, brain infection, or space-occupying lesion; (2) combined with other serious diseases (such as acute myocardial infarction, respiratory failure, kidney failure, shock, coma, or sepsis); (3) died within 7 days after onset or did not complete a 90-day follow-up were considered as dropped cases; or (4) MRI and MRA images were non-diagnostic.

### Imaging Examination

The sizes and locations of the intracranial aneurysms within 72 h after admission were determined using head MRI (Siemens Magnetom Verio 3.0 T MRI, Siemens, Erlangen, Germany) and digital subtraction angiography (DSA) (Siemens, Erlangen, Germany). The intracranial aneurysms were classified according to their size, as <3.0, 3.0–4.9, 5.0–6.9, 7.0–9.9, and ≥10 mm. They were also classified by locations, including internal carotid artery (ICA), anterior cerebral artery (ACA), anterior communicating artery (ACoA), middle cerebral artery (MCA), posterior cerebral artery (PCA), posterior communicating artery (PCoA), and vertebrobasilar artery (VB).

### Treatment

During hospitalization, the patients were treated according to the Guidelines for the Diagnosis and Treatment of Acute Ischemic Stroke in China 2014 (14), including conservative therapy, thrombolytic therapy, and endovascular interventional therapy. The conservative treatment included antithrombotic agents (aspirin, clopidogrel, and warfarin) and lipid-lowering drugs (statins).

### Data Collection and Definitions

Age, sex, history of cardiovascular disease, coronary heart disease, and stroke subtypes were extracted from the medical charts. The risk factors included hypertension, diabetes mellitus, dyslipidemia, atrial fibrillation, smoking, and alcohol. The Trial of ORG 10172 in Acute Treatment (TOAST) classification (15) was used to determine the subtype of ischemic stroke: large artery atherosclerosis (LAA), small vessel disease (SAA), cardioembolism (CE), and other types. The severity of the neurological deficits was evaluated on admission using the National Institutes of Health Stroke Scale (NIHSS) score (16).

### Outcome and Follow-Up

The outcome was the mRS score at 90 days after stroke onset. The outcome was classified into a good outcome (mRS score of 0–2 points) and poor outcome (mRS score of 3–6 points). Follow-up was performed monthly by outpatient visit, telephone, and readmission. The last follow-up was in November 2018.

### Statistical Analysis

All data were analyzed using SPSS 22.0 (IBM, Armonk, NY, USA). The continuous data conforming to the normal distribution (Kolmogorov–Smirnov test) were expressed as means ± standard deviations. When the variance was equal, the comparison between groups was analyzed using the independent sample *t*-test; otherwise, the comparison was performed using the *t*-test. The continuous data that did not conform to the normal distribution were expressed as medians (Q1, Q3) and analyzed using the Mann–Whitney U-test. Categorical data were expressed as frequencies (percentages) and analyzed using the chi-square test. After the logistic univariable analyses, the variables with *P*-values <0.1 were included in the multivariable logistic regression analysis. Multivariable logistic regression analysis (forward) was used to analyze the factors associated with UIA and outcomes. *P*-values < 0.05 were considered statistically significant.

## Results

### Baseline Data and Clinical Features

A total of 4,553 patients with acute ischemic strokes and transient ischemic attacks were included, of whom 4,335 met the inclusion criteria and 302 met the exclusion criteria (17 cases had a previous arachnoid hemorrhage, 49 had intracranial hemorrhage, 40 had other serious diseases, 75 had non-diagnostic MRI and MRA images, and 121 were lost to follow-up). Finally, 4,033 patients with ACI were included (Table 1). There were 2,413 males (59.8%) and 1,620 females (40. 2%). The mean age was 68.3 ± 11.6 years.

**Table 1:** Baseline and outcome for patients with ACI.

	**All**	**With UIA**	**Without UIA**	***P***
	**(*n* = 4,033)**	**(*n* = 260)**	**(*n* = 3,773)**	
Male, *n* (%)	2,413 (59.8)	135 (51.9)	2,278 (60.4)	0.007
Age, years, mean ± SD	68.3 ± 11.6	70.0 ± 12.8	68.2 ± 11.5	0.019
Smoking, *n* (%)	1,134 (28.1)	87 (33.5)	1,047 (27.8)	0.048
Drinking, *n* (%)	617 (15.3)	38 (14.6)	579 (15.3)	0.855
Hypertension, *n* (%)	3,352 (83.1)	227 (87.3)	3,125 (82.8)	<0.001
Diabetes, *n* (%)	1,783 (44.2)	109 (41.9)	1,674 (44.4)	0.443
Hyperlipidemia, *n* (%)	1192 (29.6)	88 (33.8)	1104 (29.3)	0.117
Atrial fibrillation, *n* (%)	562 (13.9)	39 (15.0)	523 (13.9)	0.608
Stroke, *n* (%)	1,057 (26.2)	30 (11.5)	377 (10.0)	0.112
Coronary heart disease, *n* (%)	407 (10.1)	72 (27.7)	985 (26.1)	0.574
TOAST classification, n (%)				0.071
Large artery atherosclerosis	2,271 (56.3)	166 (63.8)	2,104 (55.8)	
Small artery atherosclerosis	1,303 (32.3)	68 (26.2)	1,235 (32.7)	
Cardioembolism	410 (10.2)	23 (8.9)	387 (10.3)	
Other	50 (1.2)	3 (1.5)	46 (1.2)	
Type of medication, *n* (%)				0.061
Statins	3,449 (85.5)	149 (4.3)	3,300 (95.7)	
Warfarin	215 (5.2)	21 (9.8)	194 (90.2)	
Aspirin	3,485 (84.0)	186 (5.3)	3,299 (94.7)	
Clopidogrel	449 (10.8)	20 (4.5)	429 (95.5)	
Treatment, *n* (%)				0.069
Conservative treatment	3,852 (95.6)	250 (6.5)	3,602 (93.5)	
Thrombolytic therapy	110 (2.7)	2 (1.8)	108 (98.2)	
Endovascular intervention, n (%)	69 (1.7)	8 (11.6)	61 (88.4)	
Baseline NIHSS score, median (Q1, Q3)	3 (1, 6)	6 (4, 8)	2 (1, 5)	<0.001
Location				
Interval carotid artery		190 (73.1)		
Anterior cerebral artery		8 (3.1)		
Anterior communicating artery		9 (3.5)		
Middle cerebral artery		22 (8.5)		
Posterior cerebral artery		8 (1.9)		
Posterior communicating artery		8 (3.1)		
Vertebrobasilar artery		15 (5.8)		
Size, *n* (%)				
<3.0 mm		184 (70.8)		
3.0–4.9 mm		49 (18.8)		
5.0–6.9 mm		18 (6.9)		
7.0–9.9 mm		11 (4.2)		
≥10 mm		1 (0.4)		
Number, *n* (%)				
Single		233 (89.6)		
Multiple		27 (10.4)		
Modified Rankin scale score				0.785
0–2	2,543 (63.1)	166 (6.5)	2,377 (93.5)	
3–6	1,490 (36.9)	94 (6.3)	1,396 (93.7)	

*UIA, unruptured intracranial aneurysm; TOAST, Acute Stroke Treatment Test; NIHSS, National Institutes of Health Stroke Score*.

Of the 4,033 patients with ACI, 3,773 (93.55%) were without UIA, and 260 (6.45%) had a UIA. Compared with the patients without UIA, those with UIA showed a lower proportion of men (51.9 vs. 60.4%, *P* = 0.007), were older (70.0 ± 12.8 vs. 68.2 ± 11.5, *P* = 0.02), had a higher proportion of smokers (33.5 vs. 27.7%, *P* = 0.048), and had a higher proportion of hypertension (87.3 vs. 82.8%, *P* < 0.001) (Table 1). Eight patients with aneurysms underwent intervention. Their aneurysm was >10 mm. Two of them died during intervention due to aneurysm rupture. The six other patients recovered after the intervention.

### Univariable and Multivariable Analyses for the 90-Day Outcomes in Patients With ACI

Of the 4,033 patients with ACI, 2,543 patients (63.1%) had a good outcome, and 1,490 patients (36.9%) had a poor outcome. Among them, 3,852 (95.5%) were treated conservatively, 110 (2.7%) were treated with thrombolysis, 69 (1.7%) were treated with endovascular intervention, 3,934 (97.6%) were treated with oral aspirin or clopidogrel antiplatelet therapy after onset, and 215 (5.3%) patients received warfarin anticoagulant therapy. According to the TOAST classification, the LAA type accounted for the majority of 2,270 cases (56.3%), and the baseline NIHSS score at admission was 3 (1, 6) points. The age (*P* < 0.001), diabetes (*P* = 0.010), stroke (*P* < 0.001), and baseline NIHSS score (*P* < 0.001) were associated with the 90-day outcomes (Table 2).

**Table 2:** Univariable analyses of the factors associated with the 90-day outcomes.

	**Poor**	**Good**	***P***
	**(*n* = 1,490)**	**(*n* = 2,543)**	
Male, *n* (%)	873 (58.6)	1,540 (60.5)	0.219
Age, years, mean± SD	69.2 ± 11.5	67.8 ± 11.6	**0.009**
Smoking, *n* (%)	427 (28.7)	707 (27.8)	0.560
Drinking, *n* (%)	247 (16.6)	370 (14.6)	0.105
Hypertension, *n* (%)	1,223 (82.1)	2,129 (83.7)	0.184
Diabetes, *n* (%)	698 (46.9)	1,085 (42.7)	**0.016**
Hyperlipidemia, *n* (%)	427 (28.7)	765 (30.1)	0.338
Atrial fibrillation, *n* (%)	202 (13.6)	360 (14.2)	0.596
Coronary heart disease, *n* (%)	161 (10.8)	246 (9.7)	0.323
Stroke, *n* (%)	466 (31.3)	591 (23.2)	<**0.001**
TOAST, *n* (%)			0.398
Large artery atherosclerosis	862 (57.9)	1,408 (55.4)	
Small artery atherosclerosis	456 (30.1)	847 (33.3)	
Cardioembolism	152(10.2)	258 (10.1)	
Other	20 (1.3)	29 (1.1)	
Type of medication, *n* (%)			0.839
Statins	1,278 (85.8)	2,171 (85.4)	
Warfarin	87 (5.8)	128 (5.03)	
Aspirin	1,273 (85.43)	2,212 (86.98)	
Clopidogrel	171 (11.48)	278 (10.93)	
Treatment, *n* (%)			0.940
Conservative treatment	1,426 (95.70)	2,426 (95.40).	
Thrombolytic therapy	39 (2.61)	71 (2.79)	
Endovascular intervention	24 (1.61)	45 (1.77)	
Baseline NIHSS score, median (Q1, Q3)	3 (2, 6)	3 (1, 5)	**0.009**
UIA	94 (6.31)	166 (6.53)	0.785

*TOAST, Acute Stroke Treatment Org10172 Test; NIHSS, National Institutes of Health Stroke Score; UIA, unruptured intracranial aneurysm.*

*Variables with P values < 0.1 were included in multivariate regression*.

The multivariable analysis showed that age (OR = 1.009, 95%CI: 1.003–1.014, *P* = 0.003), diabetes (OR = 1.179, 95%CI: 1.035–1.342, *P* = 0.01), ischemic stroke history (OR = 1.451, 95%CI: 1.256–1.677, *P* < 0.001), and baseline NIHSS score (OR = 1.034, 95%CI: 1.018–1.050, *P* < 0.001) were independently associated with the 90-day outcomes in patients with ACI. The presence of incidental UIA was not associated with outcomes after ACI (Table 3).

**Table 3:** Multivariable analyses of the factors associated with the 90-day outcomes.

	**OR (95%CI)**	***P***
Age	1.009 (1.003–1.014)	0.003
Diabetes	1.179 (1.035–1.342)	0.013
Stroke	1.451 (1.256–1.677)	<0.001
Baseline NIHSS score	1.034 (1.018–1.050)	<0.001

*The variables included in the multivariable analysis: age, diabetes, stroke, and baseline NIHSS score.*

*OR, odds ratio; CI, confidence interval; TOAST, Acute Stroke Treatment Org10172 Test; NIHSS, National Institutes of Health Stroke Score; UIA, unruptured intracranial aneurysm*.

### Subgroup Analyses for the 90-Day Outcomes in Patients With ACI and UIA

Among the 260 patients with ACI and UIA, 166 had good 90-day outcomes, and 94 had a poor outcome (Table 4). Age (OR = 1.047, 95%CI: 1.014–1.081, *P* = 0.005), diabetes (OR = 2.119, 95%CI: 1.060–4.237, *P* = 0.03), ischemic stroke history (OR = 2.429, 95%CI: 1.174–5.028, *P* = 0.02), and baseline NIHSS (OR = 1.471, 95%CI: 1.315–1.646, *P* < 0.001) were independently associated with the 90-day outcomes in patients with ACI and UIA. In those patients, thrombolytic therapy was not associated with the 90-day outcomes (Table 5).

**Table 4:** Subgroup univariable analyses of the factors associated with 90-day outcomes in patients with ACI complicated with UIA.

	**Poor**	**Good**	***P***
	**(*n* = 166)**	**(*n* = 94)**	
Male, *n* (%)	94 (56.6)	41 (43.6)	**0.044**
Age, years, mean ± SD	67.4 ± 12.8	74.6 ± 11.6	<**0.001**
Smoking, *n* (%)	58 (34.9)	34 (35.9)	0.502
Drinking, *n* (%)	21 (12.7)	20 (21.3)	0.067
Hypertension, *n* (%)	143 (86.1)	84 (89.4)	0.255
Diabetes, *n* (%)	61 (36.8)	48 (51.1)	**0.025**
Hyperlipidemia, *n* (%)	54 (32.5)	34(36.2)	0.551
Atrial fibrillation, *n* (%)	24 (14.5)	15 (16.0)	0.745
Coronary heart disease, *n* (%)	15 (9.0)	19 (20.2)	**0.010**
Stroke, *n* (%)	36 (21.7)	26 (27.7)	**0.004**
TOAST, *n* (%)			**0.017**
Large artery atherosclerosis	98 (59.0)	68 (72.3)	
Small artery atherosclerosis	54 (32.5)	14 (14.9)	
Cardioembolism	12 (7.2)	11 (11.7)	
Other	2 (1.2)	1 (1.1)	
Type of medication, *n* (%)			1.423
Statins	90 (54.2)	59 (62.8)	
Warfarin	15 (9.0)	5 (5.3)	
Aspirin	113 (68.1)	73 (77.7)	
Clopidogrel	16 (9.6)	4 (4.3)	
Treatment pattern, *n* (%)			0.118
Conservative treatment	159 (95.8)	91 (96.8)	
Thrombolytic therapy	1 (0.6)	1 (1.1)	
Endovascular intervention	6 (3.6)	2 (2.1)	
Baseline NIHSS score, median (Q1, Q3)	5 (4, 6)	10 (7, 13)	<**0.001**

*TOAST, Acute Stroke Treatment Org10172 Test; NIHSS, National Institutes of Health Stroke Score; UIA, unruptured intracranial aneurysm.*

*Variables with P values <0.1 were included in multivariate regression*.

**Table 5:** Subgroup multivariable analyses of the factors associated with 90-day outcomes in patients with ACI complicated with UIA.

	**OR (95%CI)**	***P***
Male	1.209 (0.609–2.403)	0.587
Age	1.047 (1.014–1.081)	**0.005**
Diabetes	2.119 (1.060–4.237)	**0.034**
Coronary heart disease	2.258 (0.949–6.735)	0.064
Stroke	2.429 (1.174–5.028)	**0.017**
TOAST	0.870 (0.535–1.416	0.575
Baseline NIHSS score	1.471 (1.315–1.646)	<**0.001**

*The variables included in the multivariable analysis: male, age, diabetes, coronary heart disease, stroke, TOAST, and baseline NIHSS score.*

*OR, odds ratio; CI, confidence interval; TOAST, Acute Stroke Treatment Org10172 Test; NIHSS, National Institutes of Health Stroke Score; UIA, unruptured intracranial aneurysm.*

*Variables with P value < 0.05 were statistical difference*.

## Discussion

The management of incidental UIA is controversial, especially in patients with ACI. The aim of this study was to examine the patients with ACI treated at a single center over 9 years and who underwent UIA screening by 3D-TOF-MRA and to explore the factors associated with outcomes. The results suggest that the 90-day outcomes based on the mRS score were not affected by the presence of UIA in patients with ACI. Age, diabetes, ischemic stroke history, and baseline NIHSS score were independently associated with the early outcomes of patients with ACI. The presence of incidental UIA was not associated with the outcome after ACI. In patients with ACI and UIA, thrombolytic therapy was not associated with the 90-day outcomes.

Although DSA is the gold standard for the diagnosis of intracranial aneurysms, it is not routinely used due to the features of patients with traumatic and ACI and because it is an invasive procedure. As reported in the literature, UIA screening using 3D-TOF-MRA has similar sensitivity and detection rates as DSA, especially for aneurysms larger than 3 mm in diameter, while the false-positive rate is only 6.2%, and the rate of missed diagnosis is 5.3% (9, 17). Since MRI can establish the location and volume of the infarction in the early stage, MRA can be used within the same examination to complete the evaluation of the vascular level, which is of great significance for clinical diagnosis, differential diagnosis, clinical decision-making, and outcome determination. Of the 4,033 patients diagnosed with ICA and who underwent UIA screening using 3D-TOF-MRA, 115 patients underwent DSA. The diagnostic concordance rate was 97.0%, and eight patients were false-positives, consistent with the literature (9, 17). The natural incidence of UIA in the general population varies greatly, with an average of 3.2% (95% CI 1.9–5.2%). The UIA detection rate is also related to the screening methods and the subjects (17). Among the patients, female sex, age, smoking, and hypertension were associated with ACI complicated with UIA, which is consistent with the literature (18, 19). A meta-analysis analyzed 83 studies and found that the detection rate of UIA in females in the general population was 1.61 times higher than in males (20), which is comparable to the present study that the comorbidity rate of UIA in female patients with ACI was 1.62 times higher than that of males. The reason for this gender asymmetry is not clear, and it is speculated that it is related to the decline of estrogen levels in menopausal and postmenopausal women (21). Old age, high blood pressure, and smoking are risk factors for cerebral arteriosclerosis, suggesting that they are related to the common mechanism of arterial wall damage. Some authors found that ICA stenosis has a certain correlation with the occurrence of UIA (22). The proportion of patients with intracranial atherosclerosis with UIA is higher than in the general population, and intracranial artery stenosis suggests that atherosclerosis factor participates in the formation of UIA (23). As a result, the high comorbidity of intracranial aneurysms and intracranial stenosis might be explained by atherosclerosis causing morphological changes in fibrous tissue deposition and changes in blood flow patterns, resulting in weakened blood vessels, dilatation, and aneurysm formation (24, 25). On the other hand, the results did not show any difference in the incidence of UIA in each subtype of TOAST. Additional studies are necessary to examine this point.

Intravenous thrombolysis, antiplatelet, anticoagulation, and intensive lipid-lowering therapy within the window of ACI are generally accepted, but the safety of the treatments in the presence of a UIA is still controversial (9–11). It has been reported that by using conservative treatment with drugs, the incidence of recurrent ischemic events in patients is decreased, but the inflammatory cascade caused by the accumulation of platelets in the aneurysm will lead to the apoptosis of intracranial vascular endothelial cells and aneurysmal-like remodeling of the wall (26). Antiplatelet drugs such as aspirin can inhibit this process by affecting the degenerative pathological changes of the tumor wall (27), while antiplatelet drugs also reduce the rupture rate of intracranial aneurysms (28). The early management guidelines for patients with acute ischemic stroke in the United States in 2018 (29) still listed intracranial aneurysms <10 mm in diameter as a relative contraindication for intravenous thrombolysis, but more and more studies have proposed that for patients with ACI within the treatment time window, intravenous thrombolysis or arterial thrombolysis with UIA does not increase the risk of aneurysm rupture (11, 30). A recent meta-analysis suggests that the risk of cerebral hemorrhage is 17% higher than that of the control group when treated with recombinant tissue plasminogen activator (rt-PA) for intravenous thrombolysis in ACI with UIA (31). The risk of symptomatic intracranial hemorrhage increased by 70%, and the risk of subarachnoid hemorrhage increased by 13%, but all the results showed no statistically significant difference (31). Although there have been reported cases of subarachnoid hemorrhage caused by the rupture of an anterior communicating aneurysm after thrombolytic therapy in patients with ACI (32), and UIA is still listed as a contraindication for intravenous thrombolysis in the rt-PA drug insert, only 10 cases of intracranial aneurysms were found among the 105 patients with ACI who underwent thrombolysis in the present study. In addition, three patients developed cerebral hemorrhage after thrombolysis, but the bleeding site after thrombolysis was located in the infarct area, not at the aneurysm. This bleeding was likely to be a hemorrhagic transformation, not caused by an aneurysm rupture (33). In the present study, 0.8 and 3.1% of the 260 patients with UIA were treated with thrombolytic therapy and endovascular intervention, respectively, and the statistical analyses did not show that the different treatments in patients with ACI significantly impacted the 90-day outcomes. It could suggest that various treatments in the acute phase of ACI will not increase the risk of intracranial hemorrhage in those patients, and no events associated with adverse clinical outcomes were found, despite the fact that the proportion of patients with intravenous thrombolysis and endovascular intervention in this study is relatively low. Nevertheless, this will have to be confirmed in larger studies.

The American Heart Association published the UIA Management Guide in 2015 (9), arguing that the risk of aneurysm rupture is closely related to its size, location, and number. The annual rupture rates of aneurysms with diameter <5, 5–7, 7–10, 10–24, and ≥25 mm are 0.33, 3.1, 2.9, 10.2, and 33.1%, respectively (34). Another study showed that the 5-year cumulative rupture rate of aneurysms with a diameter of <7 mm and 7–12 mm in the anterior circulation was 0 and 2.6%, respectively (35). Nevertheless, in a study of acute ischemic stroke with UIA, most aneurysms were <10 mm in diameter (36). It is not clear whether patients with ICA and aneurysms >3 mm can be safely treated for their ACI. In addition, the location of the aneurysm is also related to the risk of rupture. ACoA and PCoA are the predilection sites for intracranial aneurysm rupture, and posterior circulation aneurysms are prone to rupture (37). For instance, the risk ratios for the ruptured vs. UIA in the anterior cerebral artery, ICA, and PCoA were 1.6 (1.1–2.5), 0.6 (0.4–0.9), and 2.4 (1.7–3.5), respectively (38). In the present study, the patients with aneurysms in the ICA accounted for 73.1%, the anterior circulatory system accounted for 88.1%, and the posterior circulatory system accounted for 11.9%. In this study, the proportion of the UIA in the different circulation segments in patients with ACI was similar to that of other studies. Nevertheless, there was no difference in the clinical outcomes among patients with ACI with different aneurysm locations. This might be caused by the small number of aneurysms. The TOAST classification was not associated with the outcome either.

The prevalence of UIA in patients with ACI is higher than that of the general population (12, 20), considering the fact that they have common risk factors or a common pathophysiological basis, including non-intervention and other possible intervening factors such as age, gender, high blood pressure, smoking, and diabetes, among others (39). The present study showed that the independent risk factors for the 90-day outcomes of patients with ACI complicated with UIA were age, diabetes, previous stroke history, and NIHSS score, which were the same prognostic factors as for all patients with ACI. In addition, in all patients, ACI was not associated with the outcomes. Prognostic studies on acute ischemic stroke have shown the same age trend (40) and that systemic dysfunction is bound to affect the outcomes in patients with ACI (41). In patients with ACI, there is a significant correlation between after-hospitalization blood glucose levels and post-stroke severity (42). Hyperglycemia promotes oxidative stress and inflammatory response, leading to microcirculatory dysfunction, causing blood–brain barrier damage, and cellular metabolic disorders accelerate the progression and outcomes of stroke (43). Previous epidemiological studies found that a history of a previous stroke was significantly associated with recurrent ischemic events in patients with stroke (44). Compared with the first onset of stroke, the disability rate of patients with recurrent stroke is higher, especially in elderly male patients (44). The NIHSS is widely used to assess the severity of an acute stroke and the outcomes of the patients. The higher the baseline NIHSS score, the higher the risk of poor outcomes (45). This is the same as the results of this study.

The innovation of this paper is limited. Still, its strength is that the data are from a large number of patients and can be used to support the clinical practice, especially in guiding thrombolytic therapy for patients with aneurysms, since it does not affect the final outcomes. On the other hand, there are some limitations to our study. The data were from a single-center retrospective cross-sectional observational study. All patients underwent MRA as a routine imaging examination in patients suspected of hemorrhagic stroke, but it might be seen as a selection bias since this examination is not performed routinely in all hospitals. The follow-up was only 90 days, and there was a lack of long-term data. Only 6.5% of the patients had UIAs. This is probably because the data collected for this retrospective analysis were from 2009 to 2018 and the possibility of a low positive rate of 3.0-T MRI imaging in the early period due to different criteria and radiologists with different experience evaluating the images. Especially, in the diagnosis of small aneurysms, some vascular protrusions were not included in the screening results of aneurysms in the early period of this study. In addition, there were a high proportion of small aneurysms. Although many studies have shown that the presence of small aneurysms does not increase the risk of bleeding and mortality, the current clinical work is bound to follow the guidelines, and we need larger studies and prospective randomized controlled studies for assessing the risk of aneurysmal bleeding in the context of ACI and to decide whether to adopt a positive treatment method to make patients safer and benefit more from the treatments.

In conclusion, the presence of intracranial aneurysms has no significant effect on the 90-day outcomes of patients with ACI, including the location and size of the aneurysms and different treatment methods. Thrombolytic therapy was not affected by the presence of UIA in patients with ACI. The clinical management of intracranial aneurysms must fully assess the risk of rupture and treatment, as there is no large prospective study that investigated whether early intervention for UIA can prevent acute ischemic events, and there is no evidence to support that existing therapeutic methods, including endovascular intervention and surgical treatment, can give UIA patients a better outcome. Age, diabetes, previous stroke history, and baseline NIHSS are risk factors for poor outcomes in patients with ACI and UIA, suggesting that acute cerebrovascular disease and intracranial aneurysms have a common pathophysiological basis. Therefore, physicians should pay attention to the early intervention on risk factors for cerebrovascular disease; strengthen the management of patients with advanced age, hyperglycemia, and previous stroke; and reduce the incidence of stroke and disability.

## Data Availability Statement

The raw data supporting the conclusions of this article will be made available by the authors, without undue reservation.

## Ethics Statement

This work has been carried out in accordance with the Declaration of Helsinki (2000) of the World Medical Association. This study was approved by the ethics committee of the Affiliated Hospital of Yangzhou University (approval No.2018-YKL-015). This article is a retrospective study. Therefore the Institutional waived the requirement to obtain distinct written informed consent from the patients. Written informed consent for participation was not required for this study in accordance with the national legislation and the institutional requirements.

## Author Contributions

XW, ZD, and TT: conception and design. ZD and TT: administrative support. ZD, YL, CZ, ZJ, and YZ: provision of study materials or patients. XW, YL, ZD, CZ, ZJ, and YZ: collection and assembly of data. XW, ZD, and YZ: data analysis and interpretation. All authors: manuscript writing and final approval of manuscript:.

## Conflict of Interest

The authors declare that the research was conducted in the absence of any commercial or financial relationships that could be construed as a potential conflict of interest.
